# Biodegradation of Deoxynivalenol by a Novel Microbial Consortium

**DOI:** 10.3389/fmicb.2019.02964

**Published:** 2020-01-08

**Authors:** Yanxia Wang, Gang Wang, Yijun Dai, Yu Wang, Yin-Won Lee, Jianrong Shi, Jianhong Xu

**Affiliations:** ^1^Jiangsu Key Laboratory for Microbes and Functional Genomics, Jiangsu Engineering and Technology Research Center for Industrialization of Microbial Resources, College of Life Science, Nanjing Normal University, Nanjing, China; ^2^Jiangsu Key Laboratory for Food Quality and Safety-State Key Laboratory Cultivation Base, Ministry of Science and Technology, Key Laboratory for Agro-Product Safety Risk Evaluation (Nanjing), Ministry of Agriculture and Rural Affairs, Collaborative Innovation Center for Modern Grain Circulation and Safety, Institute of Food Safety and Nutrition, Jiangsu Academy of Agricultural Sciences, Nanjing, China; ^3^Department of Agricultural Biotechnology, Seoul National University, Seoul, South Korea; ^4^School of Food and Biological Engineering, Jiangsu University, Zhenjiang, China

**Keywords:** deoxynivalenol, bacterial consortium, biodegradation, 3-keto-DON, mycotoxin

## Abstract

Deoxynivalenol (DON), a common mycotoxin of type B trichothecene, is produced mainly by several *Fusarium* species. DON causes great losses in farming and poses severe safety risks to human and animal health. Thus, DON contamination in cereals and DON toxicity are of worldwide concern. In this study, we screened the bacterial consortium C20, which efficiently degraded almost 70 μg ml^−1^ DON within 5 days. The bacterial consortium also had the ability to degrade 15-acetyl-DON, 3-acetyl-DON, and T-2 toxin. The bacterial consortium C20 was able to degrade DON under a wide range of pH and temperature conditions. The optimal temperature and pH for DON degradation were 30°C and pH 8.0, respectively. The bacterial consortium C20 comprised of different bacterial genera, and several strains were found to significantly increase when cultured in Mineral Medium with 100 μg ml^−1^ DON based on the analysis of the sequences of the hypervariable V3-V4 region of the 16S rRNA gene. 3-keto-DON was confirmed as a degradation product of DON by liquid chromatography/time-of-flight/mass spectrometry (LC-TOF-MS) and nuclear magnetic resonance (NMR) analyses. The results indicated that the bacterial consortium C20 is a potential candidate for the biodegradation of DON in a safe and environmentally friendly manner.

## Introduction

Mycotoxins have adverse effects on grain quality, resulting in economic losses, besides the threat to human and animal health. The main groups of mycotoxins in world-wide concern are aflatoxin, ochratoxin, patulin, fumonisin, trichothecene, deoxynivalenol, and zearalenone due to their toxicity and their contamination of cereals (James and Zikankuba, 2018). Among these mycotoxins, trichothecenes are mainly produced by a range of fungi, including *Fusarium, Myrothecium*, *Trichoderma*, and *Stachybotrys* (Pessu et al., 2011). Trichothecenes not only cause growth impairment and immune dysfunction at low doses but also contribute to diarrhea, emesis, leukocytosis, hemorrhage, endotoxemia, and death at higher doses (Ueno, 1983). Deoxynivalenol (DON), also known as vomitoxin, produced by *Fusarium graminearum* and *Fusarium culmorum*, is one of the most important type B-trichothecene mycotoxins (Sobrova et al., 2010). It causes acute toxicity, vomiting, anorexia, growth retardation, reproductive and teratogenic effects, skin disorders, diarrhea, carcinogenesis, and even immune dysregulation (Pestka, 2010; Sobrova et al., 2010). DON is a frequent contaminant of grain cereals, such as wheat, maize, and barley, which can be also detected in the foods of many countries (Thuvander et al., 2001; Rasmussen et al., 2003; Leblanc et al., 2005; Schollenberger et al., 2005; Castillo et al., 2008; Sugiyama et al., 2009; Alkadri et al., 2014; Stanciu et al., 2016; Shi et al., 2018). Since DON is commonly detected in grains and is chemically stable, it represents a permanent health risk to humans and domestic animals. Therefore, the development of efficient decontamination strategies is essential to DON in grains and agricultural products.

Several strategies have been developed to control DON contamination. Firstly, cultural practices may help to combat plant diseases and fungal growth, such as the multi-field rotation of crops, the selection of seeds that are resistant to *Fusarium* head blight, and the early harvest of crops (Awad et al., 2010). The suitable storage of grains after harvest can also help to avoid increased DON contamination (Shapira et al., 2004). Physical methods, such as damaged grain removal, sieving, dehulling, radiation, and adsorbent use, were successful in detoxifying DON in cereal crops (Leibetseder, 2006). A wide variety of chemical methods have also been found to be effective against DON, including heating, alkaline hydrolysis, and oxidation (Young et al., 1986; He et al., 2010).

Compared with physical and chemical detoxification, microbial biotransformation attracted considerable interests due to high degradation efficiency, the metrics of mild reaction condition, sustainable and eco-friendly process (Awad et al., 2010). Several studies have demonstrated that DON could be metabolized to lower toxicity compounds performed by several microorganisms. For instance, DON was found to be converted into 3-keto-DON under aerobic conditions by the *Agrobacterium-Rhizobium* strain E3–39, isolated from soil samples (Shima et al., 1997). Zhou et al. revealed that two metabolites of DON during degradation by *Barpee* were identified as stereoisomers of DON and 3-keto-DON (Zhou et al., 2008). In addition, microbial consortia have shown the potential to degrade DON. One mixed culture was able to convert DON into two chromatographically separable metabolites (Andrea et al., 2004). Chicken intestinal microbes were also capable of degrading DON by de-epoxidation (Young et al., 2007).

Compared with single bacterium and anaerobic microbes, bacterial consortia with ability of DON degradation under aerobic conditions are rarely reported. Moreover, the DON degradation conditions of these anaerobic bacteria were still limited. In the present study, we isolated a novel bacterial consortium C20 from the wheat field in Jiangsu Province, China, which can stably and efficiently degrade DON into 3-keto-DON under a wide range of pH and temperature with aerobic condition. Further, the performance of biodegradation of other trichothecene mycotoxins was also evaluated.

## Materials and Methods

### Samples

A total of 174 samples from different locations, including soil, rice, corn, wheat, rice panicles and fresh corn leaves, were collected during 2016–2017 in Jiangsu Province, China (Table 1). Notably, soil samples were randomly collected from the upper layers (0–30 cm) of different crop fields, such as rice, soybean, corn, and winter wheat fields.

**Table 1:** Information for samples collected during 2016–2017 in Jiangsu province.

Number	Sample	Location
A1–A3	Panicles of rice	Suqian
A4–A7	Panicles of rice	Wuxi
A8–A10	Panicles of rice	Nanjing
B1–B4	Rice	Xuzhou
B5–B11	Rice	Nantong
C1–C10	Soil	Nanjing
C11–C16	Soil	Xuzhou
C17–C25	Soil	Suqian
D1–D8	Fresh leaves of corn	Xuzhou
E9–E17	Corn	Taizhou
F1–F24	Wheat	Nanjing
F25–F48	Wheat	Taizhou
F49–F73	Wheat	Yangzhou
F74–F91	Wheat	Xuzhou
F92–F111	Wheat	Yancheng

### Culture Media and Mycotoxins

Minimal medium (MM) was used as a culture medium for the enrichment and isolation of DON-degrading strains. The composition of MM (pH 7.0) was described by Wang et al. (2019b). Ingredients were purchased from Sigma-Aldrich (Oakville, Ontario, Canada), Guanghua Sci-Tech Co., Led. (Shantou, Guangdong, China), XILONG SCIENTIFIC (Shantou, Guangdong, China), and Sinopharm Chemical Reagent Co., Ltd. (Shanghai, China). Toxin standards for DON, HT-2, T-2, 3-acetyldeoxynivalenol (3-ADON), and 15-acetyldeoxynivalenol (15-ADON) were purchased from Romer Labs (Erber Campus, Getzersdorf, Austria).

### Selection of Deoxynivaleno-Degrading Microbes

Five-gram aliquots of each sample from the 174 field samples were smashed by a pulverizer (JYL-c020E, Joyoung, China), dispersed in 50 ml distilled water, and cultured with shaking at 180 rpm for 30 min. About 500 μl of the suspension was immediately transferred to 4.5 ml MM broth media with DON (10 μg ml^−1^) as the sole carbon source. Tebuconazole and Carbendazim (1 or 10 μg ml^−1^) were added in the cultures to inhibit fungal growth, respectively. The cultures were grown at 30°C, 180 rpm. To exclude physical adsorption, samples autoclaved at 121°C for 20 min were set as negative controls. The extent of DON biotransformation was determined by high-performance liquid chromatography (HPLC). Then, the cultures with the ability of DON degradation were further inoculated on the MM broth containing 10 μg ml^−1^ DON at intervals of 7 days. To investigate DON degradation activity, the cultures were used to degrade 100 μg ml^−1^ DON. MM with DON (100 μg ml^−1^) was set as negative control. The bacterial growth was measured by optical density. The changes in DON concentration and OD _600_ values were monitored every day.

### Sample Preparation and High-Performance Liquid Chromatography Analyses

DON was extracted from fermentation broth with an equal volume of ethyl acetate for three times. After dried under an N_2_ stream, it was re-dissolved in methanol and quantified by HPLC (Waters 2695, Milford, USA) equipped with a PDA (photo-diode array) detector at a wavelength of 218 nm and an Atlantis T3 column (octadecylsilyl, ODS; 4.6 mm × 150 mm, 3 μm; Waters), as described previously (Wang et al., 2019b). The mobile phase was methanol/water (20/40, v/v) with a flow rate of 0.6 ml min^−1^ at 35°C. The identification of mycotoxin was achieved by comparing the retention times while using DON as a standard. DON and degradation products were quantified by measuring peak areas and comparing with MS calibration curves. All data are presented as mean ± standard deviation of three replicates. Degradation percentage of DON was calculated as follows:

$$D_{r}=\left(1-C_{t}/C_{ck}\right)\times 100\%$$

where *D_r_* represents the degradation percentage, and *C_t_* and *C*_ck_ are the DON concentrations in the experimental treatment and the control groups, respectively (Wang et al., 2019b).

### Analysis of Bacterial Population Diversity

To analyze the bacterial population diversity and its change at high concentrations of DON, the enriched microbial consortium C20 was isolated from environmental samples and was added to MM to degrade DON, with DON concentrations of 10 and 100 μg ml^−1^. The bacteria were incubated in media with different concentrations of DON at 30°C for 72 h. Genomic DNA of the enriched microbial cultures was extracted and purified with Gentra Puregene Yeast/Bacteria Kit (Qiagen Inc., Mississauga, Ontario, Canada). The hypervariable V3-V4 region of the 16S rRNA gene was amplified by PCR with primers 341F (CCTACGGGNGGCWGCAG) and 805R (GACTACHVGGGTATCTAATCC) (Ahad et al., 2017) and sequenced by Sangon Biotech Co. (Shanghai, China). Further, the 16S rRNA (V3-V4 region) gene sequences were compared with the ribosomal database project (RDP)^1^. All treatments were conducted in triplicates.

### Factors Influencing Deoxynivaleno Degradation

To screen for optimal degrading conditions of DON by C20, the degradation rates of DON were determined at the different conditions including temperature, pH, and bacterial concentrations. An aliquot (200 μl) of each microbial culture was added to 1.8 ml of MM supplemented with 10 μg ml^−1^ DON. The effect of incubation temperature (20, 25, 30, 35, and 40°C), medium pH (4.0, 5.0, 6.0, 7.0, 8.0, and 9.0), and inoculation size (1, 3, 5, 10, 15, and 20%) on biodegradation of DON were investigated. All treatments were performed by three independent experiments with three replicates. The optimal degradation conditions of temperature, pH, and inoculation amount were determined by the degradation rates of DON.

### Type A- and B-Trichothecene Mycotoxins Degradation

To examine the ability of C20 to transform type A- and B-trichothecene mycotoxins, which included two type A-trichothecene mycotoxins (HT-2 toxin and T-2 toxin) and three type B-trichothecene mycotoxins (DON, 3-ADON, and 15-ADON), 10 μg ml^−1^ of each mycotoxin was added to MM in 2 ml microcentrifuge tubes. Each tube was inoculated with 200 μl of C20, which was cultured in MM with 10 μg ml^−1^ DON for 72 h. Three replicate cultures were evaluated for each trichothecene mycotoxin, and the negative controls lacked C20. All cultures were incubated at 30°C and 180 rpm in a shaking incubator for 72 h, extracted three times with ethyl acetate, as described, re-dissolved in methanol and analyzed by liquid chromatography with tandem mass spectrometry (LC-MS/MS).

The LC-MS/MS system was equipped with a Shimadzu 20 AD XR (Kyoto, Japan) coupled with an AB Sciex QTRAP 3500 mass spectrometer (Foster City, CA, USA) and an XDB-C_18_ analytical column (2.1 mm × 150 mm, 3.5-μm bead diameter; Agilent) maintained at 30°C. Nitrogen was used as the drying gas. The capillary voltage was 4 kV, the nebulizer pressure was 30 psi, and the drying gas temperature was 300°C. The gradient elution conditions were methanol/water (5/95, v/v) to methanol/water (90/10, v/v) in 10 min and were held at 90% methanol for 3 min, the flow rate was set at 0.2 ml min^−1^ (Wang et al., 2019b). Analyst 1.6.1 software was used for data acquisition and processing.

### Identification of Deoxynivaleno Degradation Products

To analyze the transformation of DON, C20 was added to broth containing 10 μg ml^−1^ DON and incubated at 30°C on a rotary shaker (180 rpm) for 72 h. Culture broth containing C20 alone was used as a control. Samples were prepared as described above and detected by HPLC. The mobile phase was methanol/water (50:50, v/v) with the flow rate of 0.8 ml min^−1^.

To investigate the structure of DON degradation products, C20 was inoculated into MM medium with 100 μg ml^−1^ DON and cultured for 72 h at 30°C with shaking at 180 rpm. The DON degradation products were extracted three times with ethyl acetate. The organic phase was pooled, concentrated and applied to a Waters 1525 Prep-HPLC system (Milford, MA, USA) equipped with a UV detector. Each 20 μl extract was injected onto an XBridge™ Prep C_18_ column (19 mm × 100 mm, 5 μm, Waters) with methanol/water (50:50, v/v) at a flow rate of 2 ml min^−1^. DON and its degradation products were detected at a wavelength of 218 nm at 35°C. The resulting degradation products were dried under an N_2_ stream and re-dissolved in CDCl_3_ for nuclear magnetic resonance (NMR) analysis.

The ^1^H NMR spectra of the biodegradation products were measured by a Bruker DRX-600 spectrometer operated at 600 MHz. The chemical shift (δ) was recorded in parts per million (ppm) relative to the solvent signals [δ(H) 7.26], and the coupling constant (*J*) was measured in Hz.

### Data Analysis

Data were organized using Microsoft Excel 2010. All values are expressed as the mean of three replicates ± standard deviation. The mean differences among treatments were considered significantly at *p* < 0.05 and were evaluated with Tukey’s-honestly significant difference (HSD) test (Ahad et al., 2017).

## Results

### Isolation of Deoxynivaleno-Degrading Bacteria

**Figure 1:**
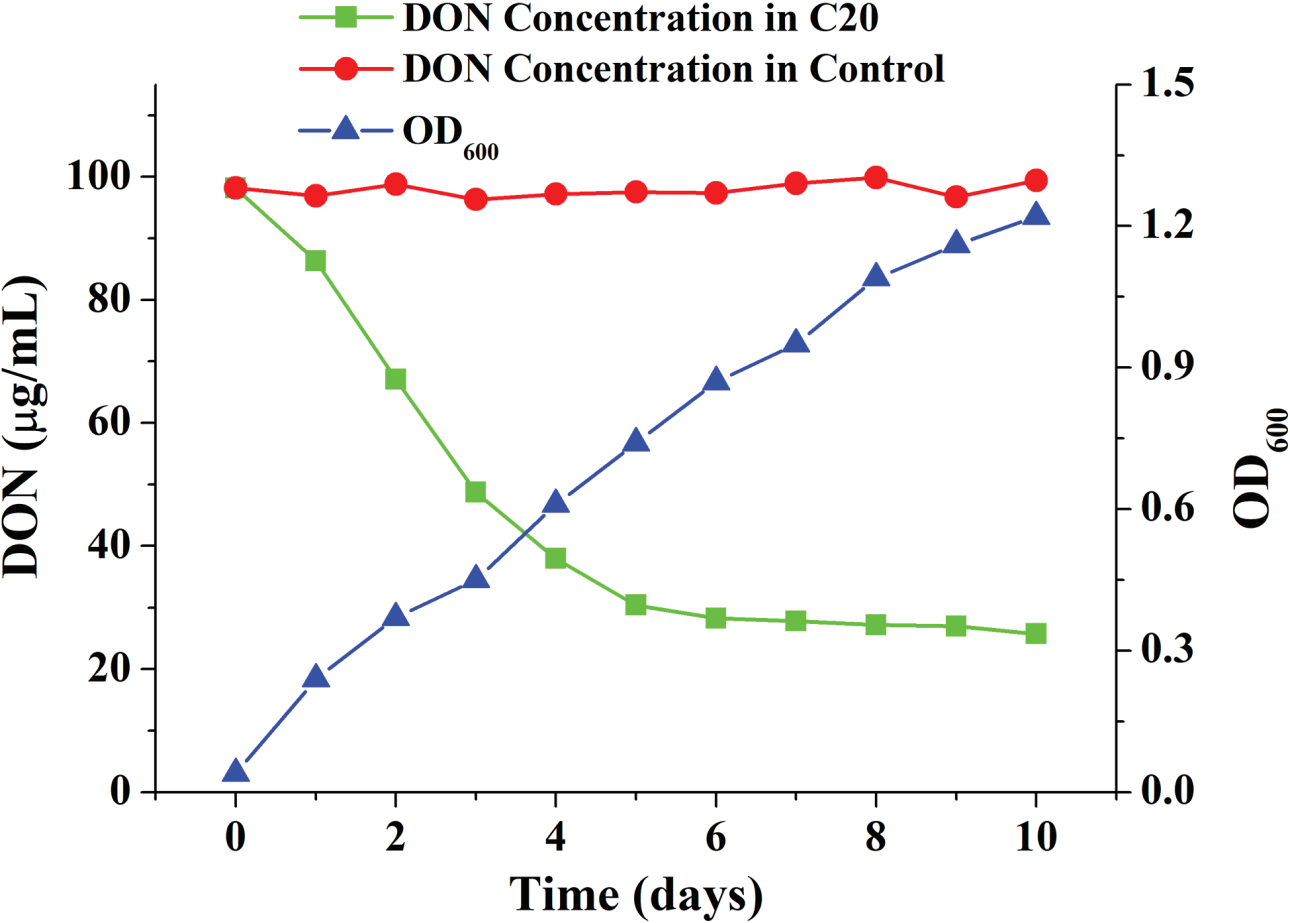
Growth and DON-degrading efficiency of the enriched culture C20.

To screen for the microbes that degraded DON, DON was used as the sole carbon source for the enrichment and isolation of the mixed culture. The mixed microbial culture C20, isolated from a soil sample from Suqian, degraded 74.29 μg ml^−1^ DON after incubation at 30°C for 10 days. DON was degraded rapidly within 5 days of incubation and was continuously degraded after incubation for 10 days. The biomass increased continuously with the amount of DON decreased (Figure 1). Furthermore, no difference was found between the samples with and without fungal inhibitors. And the DON concentration in the autoclaved sample was not reduced (Supplementary Figure S5). Importantly, the enriched bacterial consortium C20 could efficiently degrade DON, even after more than 100 subcultures in broth (Supplementary Figure S3).

### Bacterial Diversity Analysis of the Microbial Consortium C20

**Figure 2:**
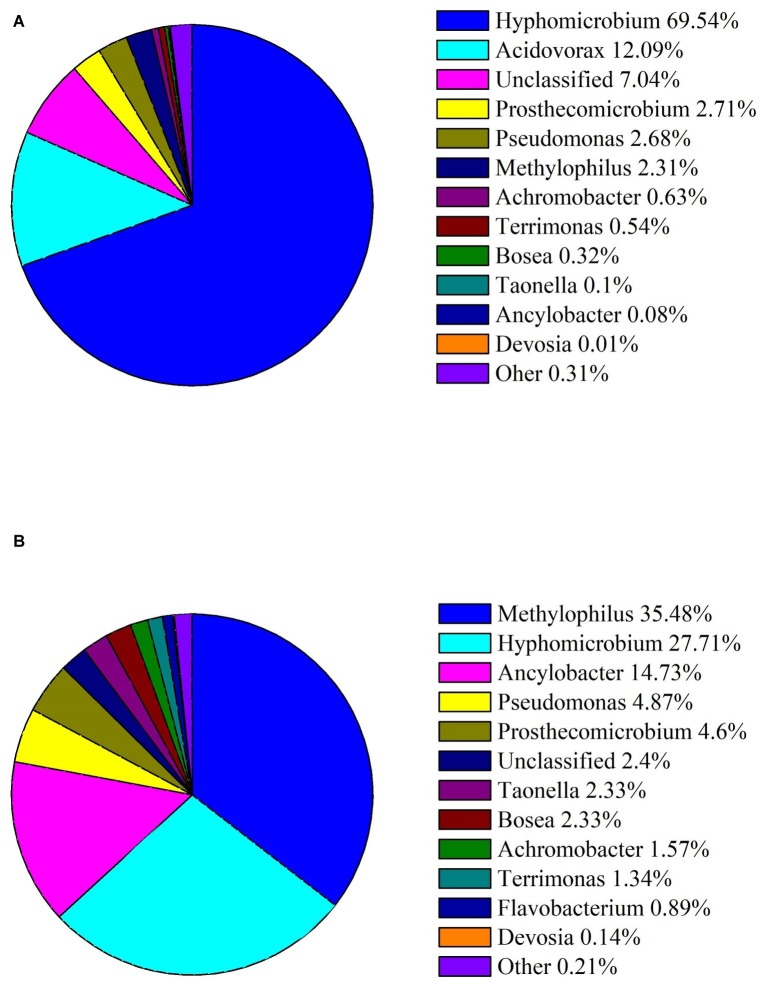
Abundances of bacterial genera in the enriched culture C20. **(A)** Relative abundance of bacterial genera in C20 in MM with 10 μg ml^−1^ DON. **(B)** Relative abundance of bacterial genera in C20 in MM with 100 μg ml^−1^ DON. The different colors and areas represent different bacterial genera and their relative abundance present in C20.

To examine mainly bacteria involved in DON degradation, the microbial diversity dynamics of C20 with different concentrations of DON was defined through analyzing the sequences of the 16S rRNA genes, as described in methods. The results showed that the enriched bacterial consortium C20 mainly consisted of the genera *Hyphomicrobium* (69.54%), followed by *Acidovorax* (12.09%), unknown microbes (7.04%), *Prosthecomicrobium* (2.71%), *Pseudomonas* (2.68%), and *Methylophilus* (2.31%) in MM with 10 μg ml^−1^ DON (Figure 2A). In contrast, the culture in MM with 100 μg ml^−1^ DON contained a heterogeneous community composed of *Methylophilus* (35.48%), followed by *Hyphomicrobium* (27.71%), *Ancylobacter* (14.73%), *Pseudomonas* (4.87%), *Prosthecomicrobium* (4.6%), unknown microbes (2.39%), *Taonella* (2.33%), *Bosea* (2.33%), and others (Figure 2B). Noticeably, as the DON concentration increased to 100 μg ml^−1^, there was a marked increase in *Methylophilus*, *Ancylobacter*, *Pseudomonas, Prosthecomicrobium, Taonella*, *Bosea*, *Terrimonasin*, and *Devosia* in C20.

### Factors Influencing Deoxynivaleno Degradation

**Figure 3:**
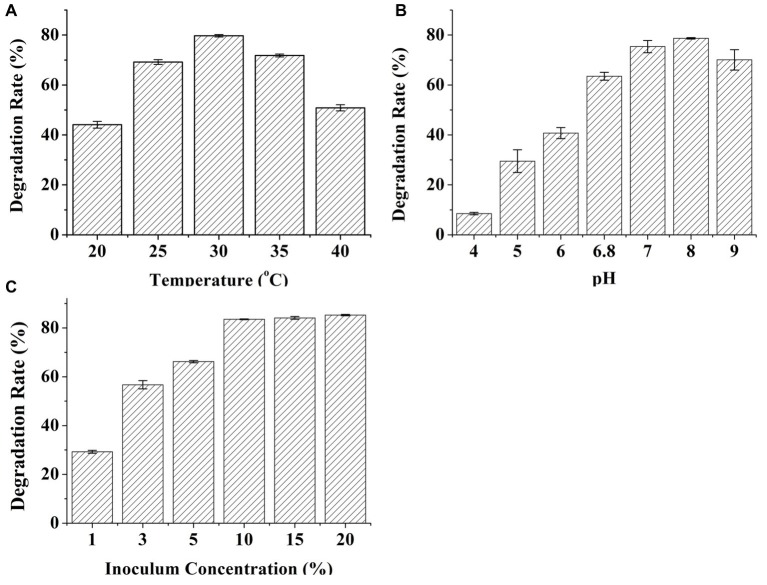
Effects of temperature, pH, and inoculum concentrations on the degradation of DON by C20. **(A)** The effect of incubation temperature on the degradation of DON. **(B)** The effect of pH on the degradation rate of DON. **(C)** The effect of inoculum concentrations on the degradation of DON. Results are the mean of five replicate observations, and bars shown are ± standard errors of the means.

The effects of the temperature, pH, and inoculum on DON degradation by C20 were investigated (Figure 3). DON degradation activity was examined at various temperatures from 20 to 40°C in the enriched cultures (Figure 3A). The highest degradation rate occurred at 30°C and decreased only slightly at 25 and 35°C. Importantly, approximately 50% of DON degradation was observed even at 40°C, indicating that the consortium C20 can effectively degrade DON over a wide range of temperatures (25–35°C). Moreover, the effects of pH on DON degradation was detected under six different pH levels conditions. The degradation rate of DON increased markedly from pH 4.0 to 8.0. The maximum DON degradation rate was observed at pH 8.0, and the degradation for 10 μg ml^−1^ DON at pH 9.0 was reduced to 70.08% (Figure 3B). The inoculum size significantly affected the degradation of DON. The degradation rates of 10 μg ml^−1^ DON after 72 h of incubation increased from 30 to 85% when the inoculum size was raised from 1 to 10% (Figure 3C).

### Degradation of Type A- and B-Trichothecene Mycotoxins by Consortium C20

**Figure 4:**
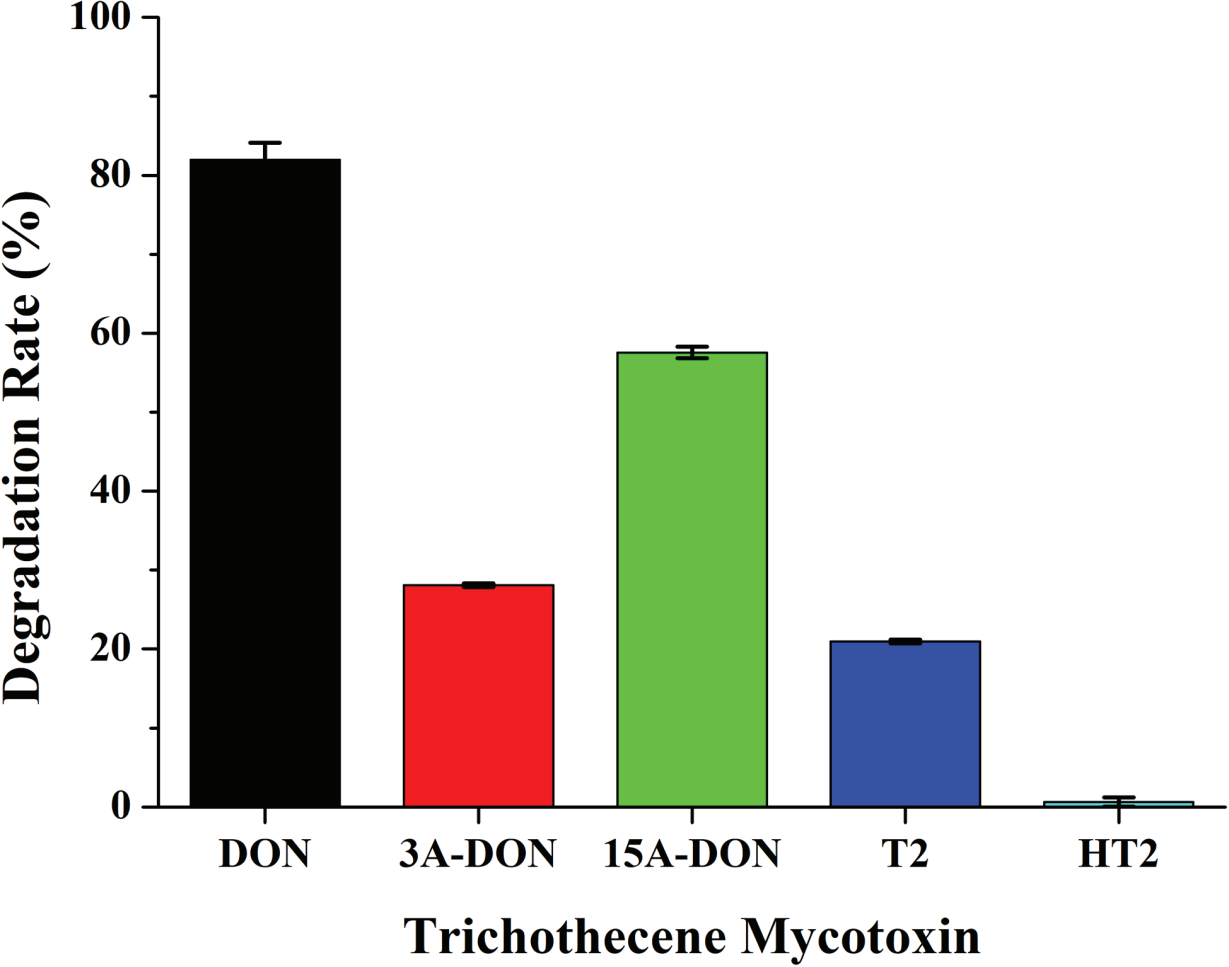
Degradation rate of DON, 3-ADON, 15-ADON, T-2, and HT-2 by the enriched culture C20.

To investigate the degradation of other trichothecene mycotoxins by C20, the consortium C20 was incubated with T-2, HT-2, 3-ADON, and 15-ADON for 72 h. The results demonstrated that the consortium C20 degraded 58% of 15-ADON, 28% of 3-ADON, and 21% of T-2. However, the degradation of HT-2 was not observed (Figure 4). 15-ADON and 3-ADON are the main acetylated derivatives of DON, and the degradation of 15-ADON and 3-ADON indicates that C20 possesses the ability to degrade acetylated DON derivatives (Payros et al., 2016).

### Identification of Deoxynivaleno Degradation Products

**Figure 5:**
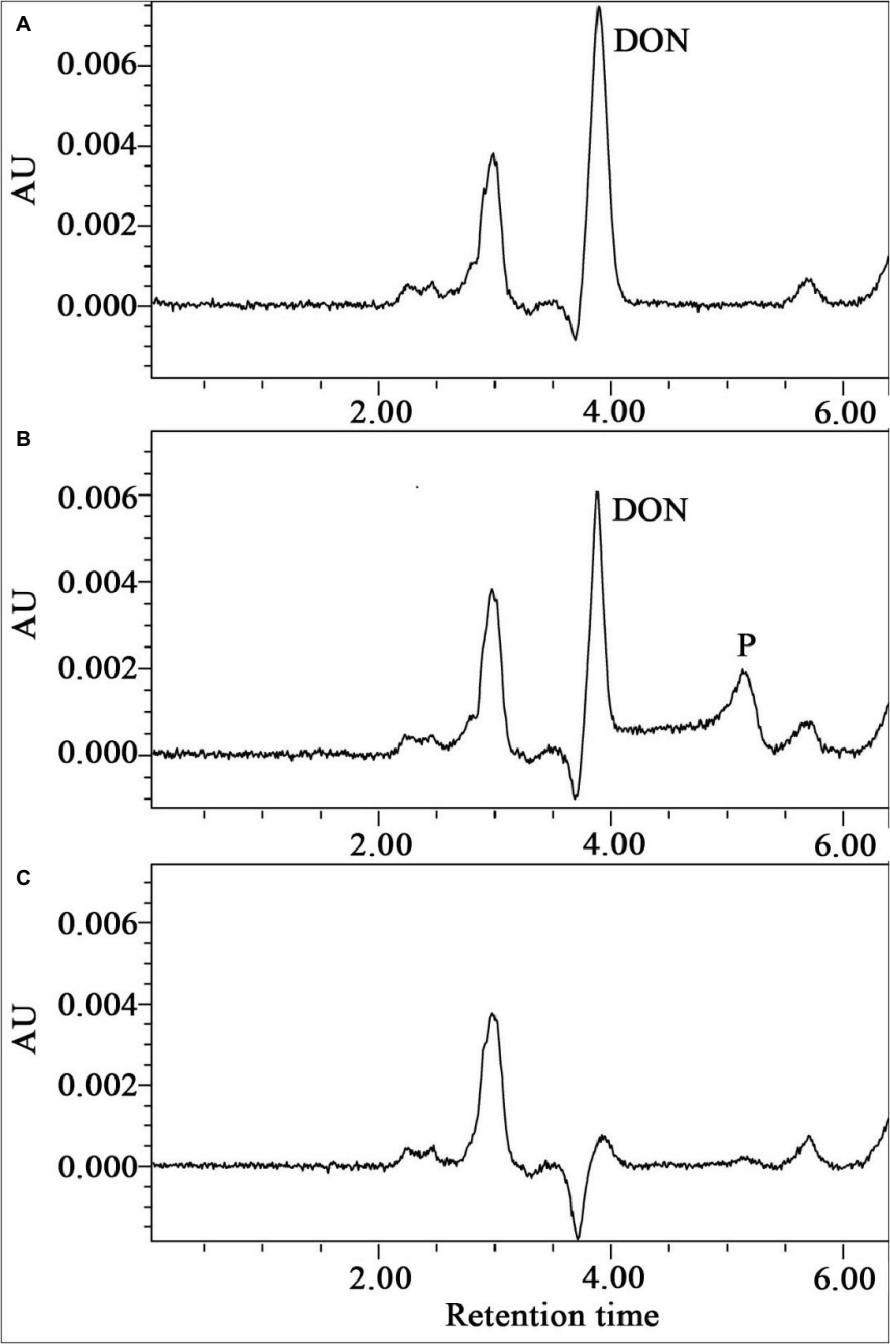
The HPLC spectra of the metabolism of DON by the enriched culture C20. **(A)** Substrate control broth containing the substrate DON alone. **(B)** Broth containing DON and C20. **(C)** Culture control broth containing C20 alone.

The metabolic products of DON were identified by using HPLC, LC-TOF-MS, and NMR. HPLC analysis showed that DON was transformed to another metabolite during degradation by the enriched bacterial consortium C20 (Compound P, Figure 5). LC-TOF-MS results (Supplementary Figure S1) showed that the molecular ion of the metabolite was at *m/z* 295.1151 ([M + H]^+^, C_15_H_19_O_6_^+^; calcd. 295.1182), which indicated a di-dehydrogenation of the DON molecule. The ^1^H-NMR spectrum for compound P (Supplementary Figure S2) was 6.55 (dd, J = 6.0, 1.5), 4.91 (d, J = 1.3), 4.57 (d, J = 6.0), 3.91 (d, J = 11.6), 3.81 (d, J = 1.5), 3.75 (d, J = 11.6), 3.52 (s), 3.36 (d, J = 4.2), 3.23 (d, J = 4.2), 3.13 (d, J = 19.5), 2.29 (d, J = 19.5), 1.90 (3H, s), and 1.34 (3H, s). The chemical formula and the ^1^H-NMR signals of compound P were identified as 3-keto-DON (Hassan et al., 2017; Wang et al., 2019b).

## Discussion

DON is one of the most commonly detected contaminants of crops in worldwide, such as maize, wheat, and rice (Lee and Ryu, 2015). The widespread occurrence of this mycotoxin, coupled with the detriments to human health and animal productivity resulting from its consumption, has promoted intensive efforts to identify detoxification measures, including microbial biotransformation (Pestka, 2010; Sobrova et al., 2010; Kris et al., 2014). Although several single microbial strains for the degradation of DON were isolated, there are still many limitations to application for agricultural fields because of the diversity and complexity of crop cultivation and storage environments (Brenner et al., 2008). Fortunately, microbial consortia provide a promising application for mycotoxin decontamination owing to their special advantages in the degradation of complex compounds. Microbial consortia combine multiple metabolic capacities of different species, improving the ability and efficiency of complex substrates biotransformation process, as well as the tolerance of complex environments (Shong et al., 2012). To date, microbial consortia has been widely used for a variety of important processes, such as the treatment of wastewater (Daims et al., 2006), the biosynthesis of compounds for medicine and industry (Shong et al., 2012), the biodegradation of bispyribac-sodium and phenanthrene (Janbandhu and Fulekar, 2011; Ahmad et al., 2018), and the production of hydrogen (Zhang et al., 2017), due to their high adaptability and broad substrate spectra (Kleerebezem and Van Loosdrecht, 2007). However, few attempts have been made to degrade DON by a microbial consortium. In this study, the highly enriched, stable and effective microbial consortium C20 was found to be able to degrade DON, resulting in a significant reduction in DON (Figure 1). In addition, tebuconazole and carbendazim have no effects on the degradation of DON by the enriched culture, indicating that the degradation of DON is owing to bacteria. Furthermore, there was no change in DON concentration in the autoclaved sample, suggesting that the soil sample cannot detoxify DON by physical adsorption (Supplementary Figure S5).

The characterization of DON degradation in this study showed the activity of the microbial culture at a wide range of pH (6.0–9.0) and temperatures (25–35°C) compared with previously studied microbial cultures (Guan et al., 2009; Wang et al., 2019b). The microbial consortium C20 might degrade DON in complex and diverse environments. The optimal pH (8.0) and temperature (30°C) of the microbial consortium C20 for DON degradation are similar to that of most arable plants. It has been suggested that microbial consortia have potential applications for the detoxification of DON in field crops (Karlovsky, 1999; Islam et al., 2012).

The functional and stability of microbial consortium are usually modulated through interactions among microorganisms. These interactions can be indirect, such as exchanging different metabolites and information signals (Wintermute and Silver, 2010). For example, *Bacillus cereus* A1 and *Brevundimonas naejangsanensis* B1 mutually cooperate to enhance hydrogen production and starch utilization. In this consortium, strain A1 produced lactate as carbon source for strain B1 to cell growth and hydrogen production. In return, formate produced by strain B1 as an electron shuttle for strain A1 to generate hydrogen (Wang et al., 2019a). In direct interactions, microorganisms exchange electrons or deliver macromolecules such as DNA and proteins by direct interspecies contact. Rotaru et al. reported that besides H_2_ or formate as the electron donor for interspecies electron transfer, direct electrical connections was also found in the production of methane between *Geobacter metallireducens* and *Methanosaeta harundinacea* (Rotaru et al., 2014).

As the sole carbon, DON provided more substrate to the microbial consortium C20 in MM with 100 μg ml^−1^ DON than in the original culture. The increasement of DON might enhance the proportion of DON-degraders during the consecutive enrichment, resulting in a change in the community composition (Ahad et al., 2017). Strains belonging to *Methylophilus*, *Ancylobacter*, *Pseudomonas, Prosthecomicrobium, Bosea*, *Taonella* and *Devosia* were significantly increased (*p* < 0.05, one-way ANOVA) in C20 after enrichment with 100 μg ml^−1^ DON. These results suggest that the changes in these bacterial populations might be related to the microbial degradation of DON. It was demonstrated that many bacteria isolated for the degradation of DON were identified as *Devosia* strains (Sato et al., 2012; Onyango et al., 2014; Yin et al., 2016; Zhao et al., 2016; Hassan et al., 2017). Moreover, DON was transformed by these bacteria to 3-epi-DON, 3-keto-DON, or other products. Therefore, the *Devosia* strains in C20 have great potential for DON degradation.

The 3-OH group in DON, as well as the epoxide group on the trichothecene backbone, is responsible for its toxicity. When DON was epimerized or degraded, its interaction with ribosome will be changed, leading to an absence of MAP kinase activation and toxicity reduction. It was reported that the intestinal toxicity of DON would be decreased when it was converted into depoxy-DON or 3-epi-DON by microbial transformation (Pierron et al., 2016). In this study, 3-keto-DON was found to the main degradation product of DON analyzed by HPLC, LC-TOF-MS and NMR. It is all known that the oxidation of DON to 3-keto-DON plays a vital role in the detoxification of DON-contaminated grains (Shima et al., 1997). In addition, the 3-keto-DON showed a reduction of more than 90% in immunosuppressive toxicity compared with that of DON (He et al., 2015).

A DON degrading bacterial strain was isolated from the consortium C20, but the strain did not maintain its DON degradation activity after a few generations of culture in broth medium (Supplementary Figure S4). It is still challenging to isolated DON-degrader through selective enrichment procedures because the degradation reaction involves the removal of an oxygen molecule (Islam et al., 2012; Yoshizawa et al., 2014), which does not supply any nutrients for bacteria (Horvath, 1972; Fedorak and Grbić Galić, 1991) and prevents the utilization of DON as the sole carbon or nitrogen source for the selective isolation of degrader from the consortium C20 (Ahad et al., 2017).

The enriched culture C20 had the ability to degrade 15-ADON, 3-ADON and T-2, as well as DON in this study. Previous studies demonstrated that 15-ADON is higher toxicity than DON or 3-ADON (Pinton et al., 2012; Kadota et al., 2013). Moreover, both DON and 15-ADON are frequently found in wheat and other cereals (Han et al., 2014; Dong et al., 2016). Thus, the microbial consortium C20 has great potential to degrade DON and 15-ADON in agricultural products.

In conclusion, the enriched bacterial consortium C20, which can efficiently degrade DON and its derivatives 15-ADON and 3-ADON through the combined strengths of individual organisms, was isolated from environmental samples in this study. C20 is comprised of different bacterial genera, with *Methylophilus*, *Ancylobacter,* and *Devosia* significantly increasing in cultures with high concentrations of DON. Importantly, about 70 μg ml^−1^ DON could be degraded by consortium C20 within 5 days under a wide range of pH and temperature conditions. And the final degradation product was identified as 3-keto-DON with less toxicity. These results suggest that the C20 microbial consortium provides a new potential method for DON degradation. Extending DON degradation capabilities from single bacteria to microbial consortia represents a new frontier in DON microbial biotransformation. Although stable community behavior remains a great challenge in this area, it still has great potential to be used to degrade DON in food/feedstuffs and to improve food/feed quality and safety.

## Data Availability Statement

All datasets generated for this study are included in the article/Supplementary Material.

## Author Contributions

YaW performed experiments and wrote the paper. GW, YuW, and Y-WL designed and performed experiments. JX, JS, and YD designed experiments, supervised the students, and wrote the paper. All authors have approved the final version of the manuscript.

### Conflict of Interest

The authors declare that the research was conducted in the absence of any commercial or financial relationships that could be construed as a potential conflict of interest.

## Footnotes

^1^http://rdp.cme.msu.edu/misc/resources.jsp
