# Supplementary material for: Biodegradation of Deoxynivalenol by a Novel Microbial Consortium
Source: Front Microbiol. 2020 Jan 8;10:2964. doi: 10.3389/fmicb.2019.02964 (PMC6960266; doi:10.3389/fmicb.2019.02964)

**Figure S1**. LC-MS/MS spectrum of DON degradation product by the enriched culture C20.


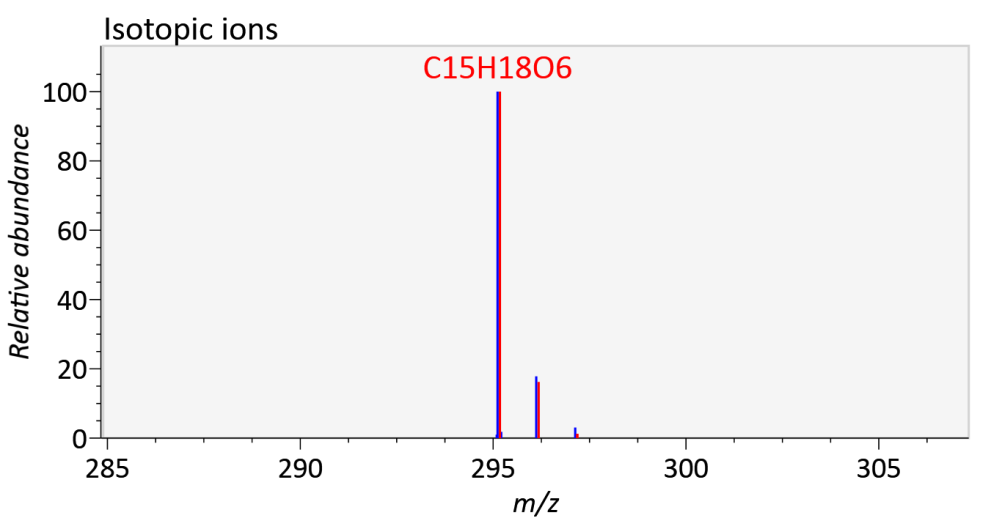


**Figure S2**. ^1^H-NMR spectrum (600 MHz) of DON degradation product by the enriched culture C20 in CDCl_3_.


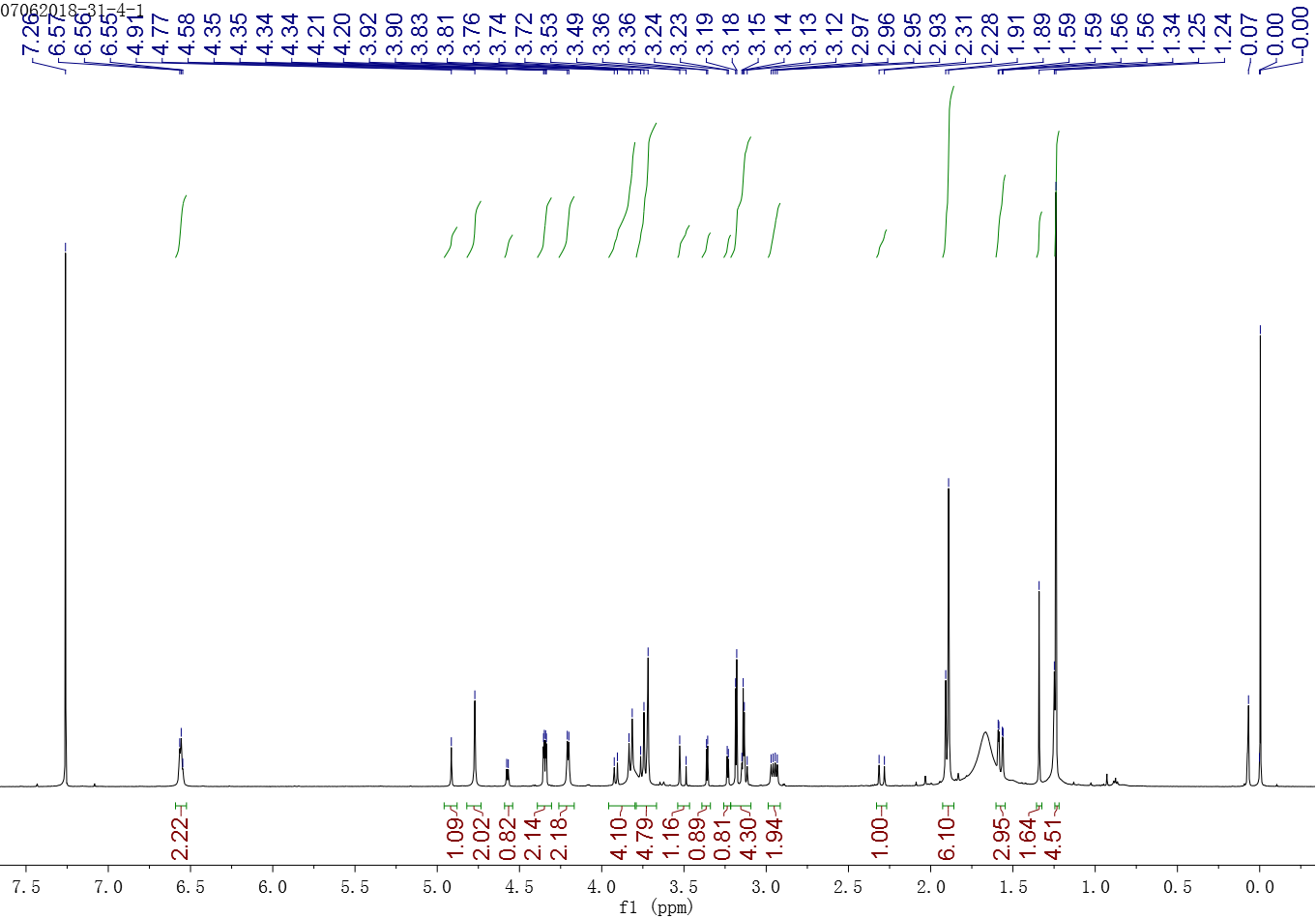


**Figure S3**. Degradation of DON by the enriched culture C20 in different subcultures.


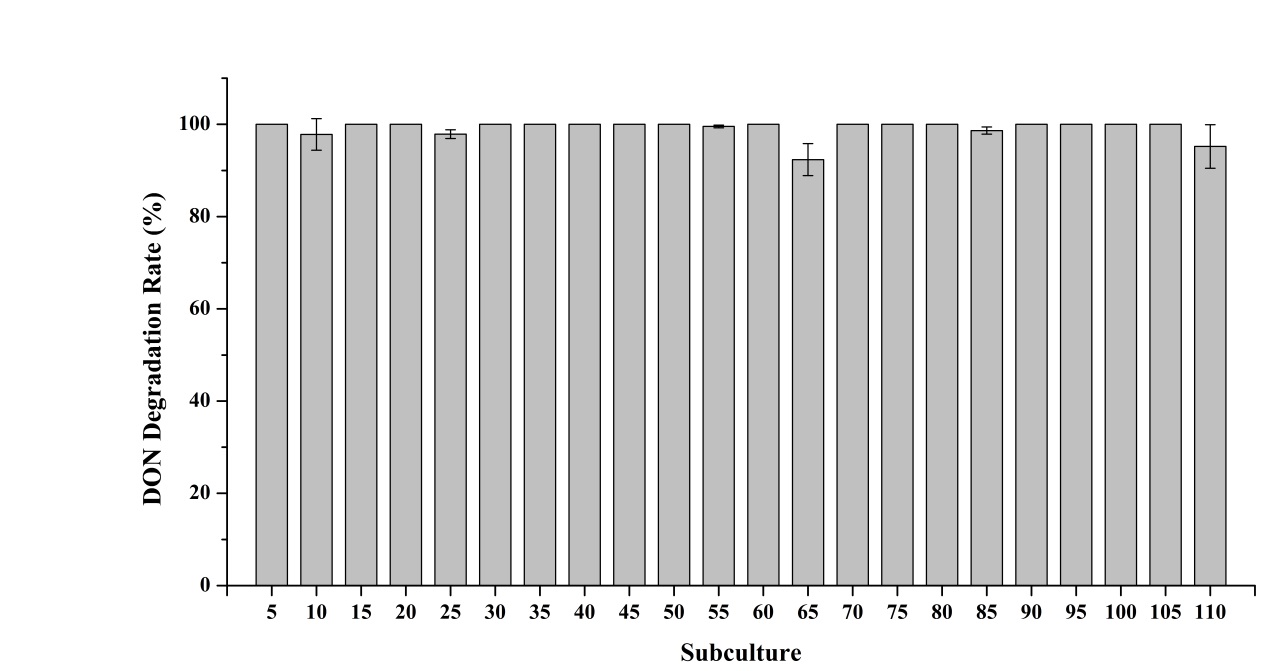


**Figure S4**. Degradation of DON by a single bacterial strain isolated from the consortium C20 in different generations.


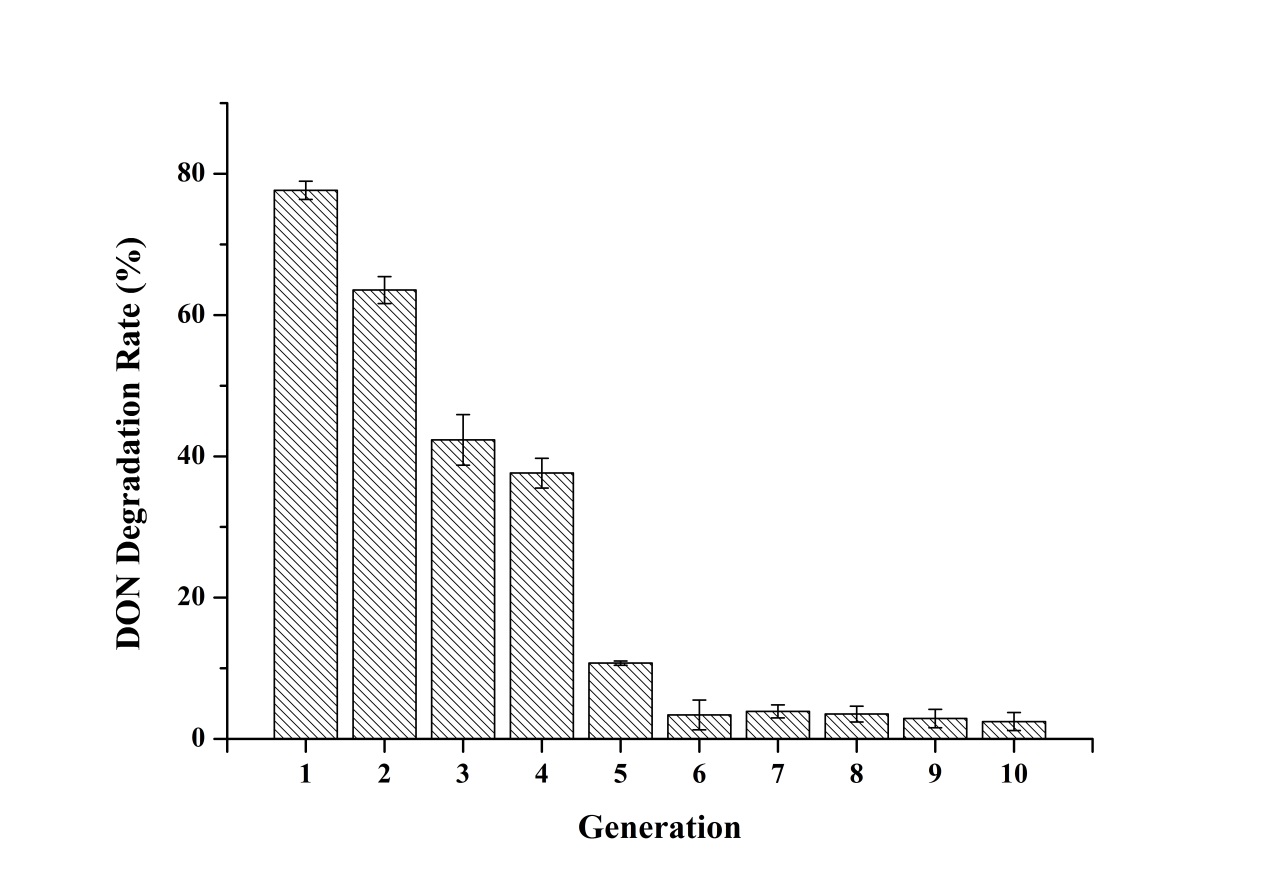


**Figure S5**. Effects of fungal inhibitor and autoclaved on the degradation of DON by C20.


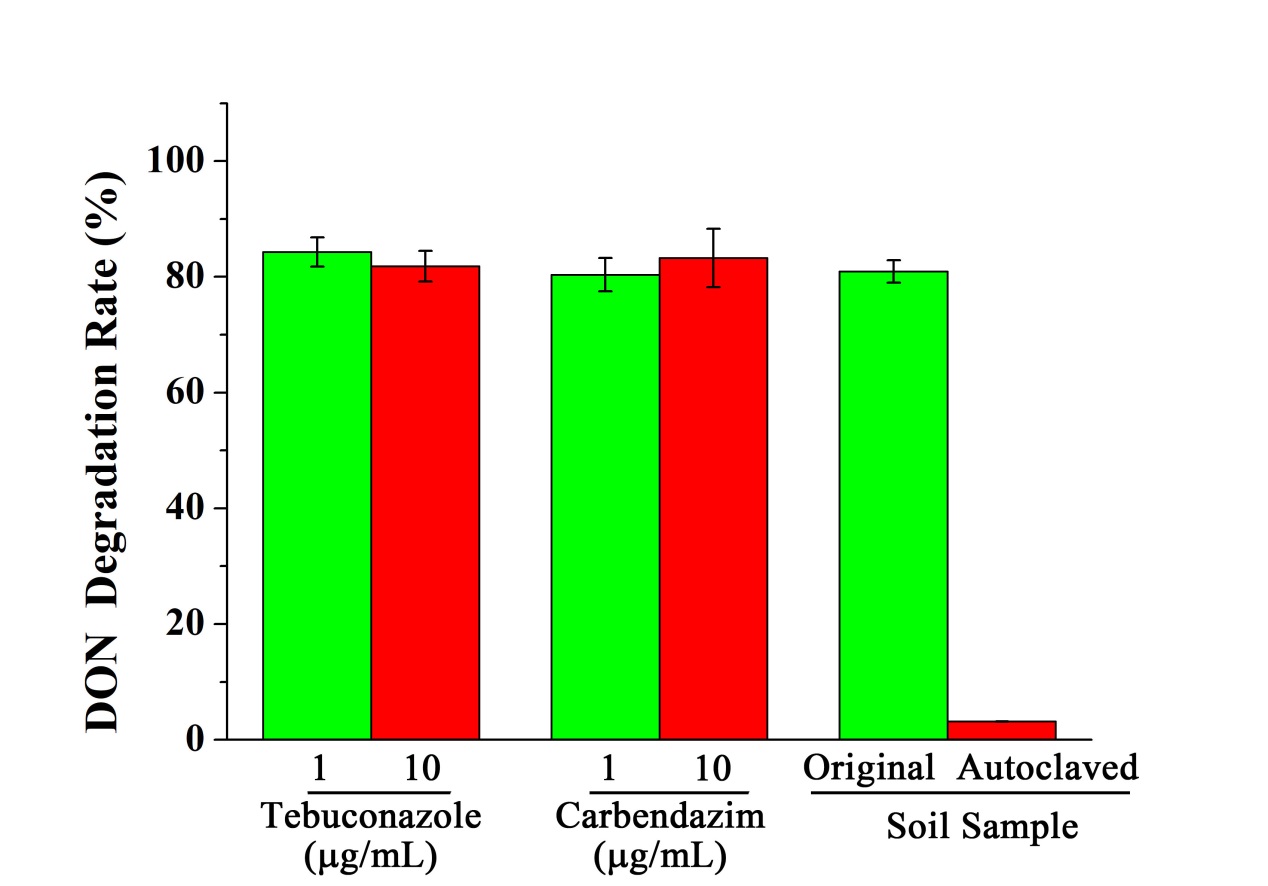

Supplement: Supplementary file 1 [file Data_Sheet_1.docx]
